# Editorial: Gastrointestinal and Liver Effects of Fruits and Their Synergism With Drug Therapy: Exploring Possible Mechanisms of Action

**DOI:** 10.3389/fphar.2022.940668

**Published:** 2022-07-01

**Authors:** Almir Gonçalves Wanderley, Lucindo José Quintans Júnior, Henrique Douglas Melo Coutinho, Jackson Roberto Guedes da Silva Almeida, Irwin Rose Alencar De Menezes

**Affiliations:** ^1^ Department of Pharmaceutical Sciences, Institute of Environmental, Chemical and Pharmaceutical Sciences, Universidade Federal de São Paulo—UNIFESP, São Paulo, Brazil; ^2^ Department of Physiology, Federal University of Sergipe—UFS, Aracaju, Brazil; ^3^ Department of Chemical Biology, Regional University of Cariri—URCA, Crato, Brazil; ^4^ Department of Pharmacy, Federal University of Vale do São Francisco—UNIVASF, Petrolina, Brazil

**Keywords:** mechanisms of action, fruits, gastrointestinal tract, liver disease, gastrointestinal disease, synergism, biological activity

Historically, natural products have been rich sources of molecules that can be used in the prevention or treatment of different pathological conditions. With the advent of the second industrial revolution and the development of organic chemistry and pharmacology, drugs of synthetic origin started being produced and used on a large scale, and products of natural origin began to occupy a secondary role in the therapeutic arsenal. However, the increase in the incidence of adverse effects and the high cost of synthetic drugs are among the factors that contributed to the rediscovery of products from biodiversity.

This Research Topic aims to add contributions to the potential use of bioactive natural products or their association with synthetic drugs in the prevention and/or treatment of liver and gastrointestinal system disorders.

This Research Topic presented one systematic review with a meta-analysis dedicated to dyslipidemia (Carvalho et al.), two experimental studies (Zhang et al.; Cui et al.), and one review study (Yu et al.) addressing Nonalcoholic Fatty Liver Disease (NAFLD). Two experimental studies explored the theme of liver fibrosis (Zhao et al.; Zhang et al.) while studies also looked at severe acute pancreatitis (Yang et al.), hepatic ischemia/reperfusion (Chen et al.), and gastroprotection (Mahmoud et al.).

Rhein is an anthraquinone derivative found in some medicinal plants, such as Rhubarb, Polygonum multiflorum, Ruta graveolens, and Folium sennae, that has hepatoprotective, nephroprotective, anti-inflammatory, antioxidant, anticancer, and antimicrobial properties (Zhou et al., 2015). Yang et al. were able to show that Rhein was effective in protecting against the deleterious effects of Severe Acute Pancreatitis (SAP) in both *in vivo* and *in vitro* models, possibly via the JAK2/STAT3 signaling pathway.

According to Younossi et al. (2018), the global prevalence of NAFLD is increasing and is estimated to be ∼25% in the general population. Yu et al. contributed to this Research Topic with a review of the underlying mechanisms of dietary fructose and its role in the development and progression of NAFLD, and they also proposed possible targets to prevent the pathogenic process. Zhang et al. reported that Tiaoganquzhi Decoction, a traditional Chinese herbal formulation, has a protective action against NAFLD, whose mode of action is associated with the promotion of CGI-58 to inhibit the expression of ROS-induced NLRP3 inflammasome. Pogostemon cablin (Blanco) Benth/Huo Xiang (HX) is a species that has antioxidant and anti-inflammatory properties with used in traditional Chinese medicine. Cui et al. explored the potential mechanism of HX on NAFLD through network pharmacological and experimental studies. The data showed that HX can attenuate abnormal lipid metabolic responses and enhance antioxidant mechanisms through multiple pathways.

Dihydromyricetin (DHM), also known as ampelosin, is a natural compound with distinct pharmacological properties and has multiple hepatoprotective effects (Chen et al., 2021). Zhao et al. investigated the effect of DHM as a novel nutritional supplement in the thioacetamide (TAA)-induced liver fibrosis model. The results showed that DHM mitigated TAA-induced hepatic fibrosis through inhibition of NF-κB-mediated inflammation and the TGF-β1-regulated PI3K/Akt signaling pathway, indicating that the compound is a potential hepatoprotective factor with prospects to be used in the treatment of liver fibrosis.

Pentagalloyl glucose (PGG) is a polyphenolic compound with potent antioxidant action and antimicrobial, anti-viral, anti-diabetic, anti-inflammatory, and antitumor properties (Patnaik et al., 2019). Mahmoud et al. evaluated the gastroprotective potential of PGG against indomethacin-induced ulcers in rats and found that this polyphenol promoted an antiulcerogenic effect mediated by increasing mucus production, scavenging free radicals, decreasing inflammation, and attenuating the NO/NOS signaling in favor of eNOS.

Kaempferol (KAE) is a flavonoid present in several plant species, including Penthorum chinense, and is traditionally used in Chinese medicine for the treatment of liver diseases (Wang et al., 2020). Chen et al. confirmed the hepatoprotective effect of KAE in the hepatic ischemia/reperfusion injury model in mice whose mechanism is associated with inhibition of oxidative stress and inflammation by activating the Nrf2/HO-1 signaling pathway.

The beneficial effects of Bone Marrow Mesenchymal Stem Cells (BMSCs) (Watanabe et al., 2021) and ferulic acid (FA) (Mu et al., 2019) in the treatment of cystic fibrosis have been described in the literature. Considering this information, Zhang et al. have expertly demonstrated the beneficial and synergistic effect of the combined therapy BMSCs and FA in a CCL4-induced liver fibrosis rat model.

Carvalho et al. have suggested in a systematic review with meta-analysis that Citrus species extracts are potential candidates for dyslipidemia control; however, the high heterogeneity indicates that more studies are required to increase the strength of this occurrence.

In summary, the different herbal formulations or compounds obtained from the plant were potentially effective in the treatment of pathological conditions that affect the liver and gastrointestinal system. We hope that these contributions serve as a guide for new pre-clinical and clinical studies, expecting to find new drug/phytopharmaceutical candidates for therapeutic use in the future.
